# Supplementary figures and images for: Molecular analysis of oral microflora in patients with primary Sjögren’s syndrome by using high-throughput sequencing
Source: PeerJ. 2018 Sep 28;6:e5649. doi: 10.7717/peerj.5649 (PMC6166617; doi:10.7717/peerj.5649)

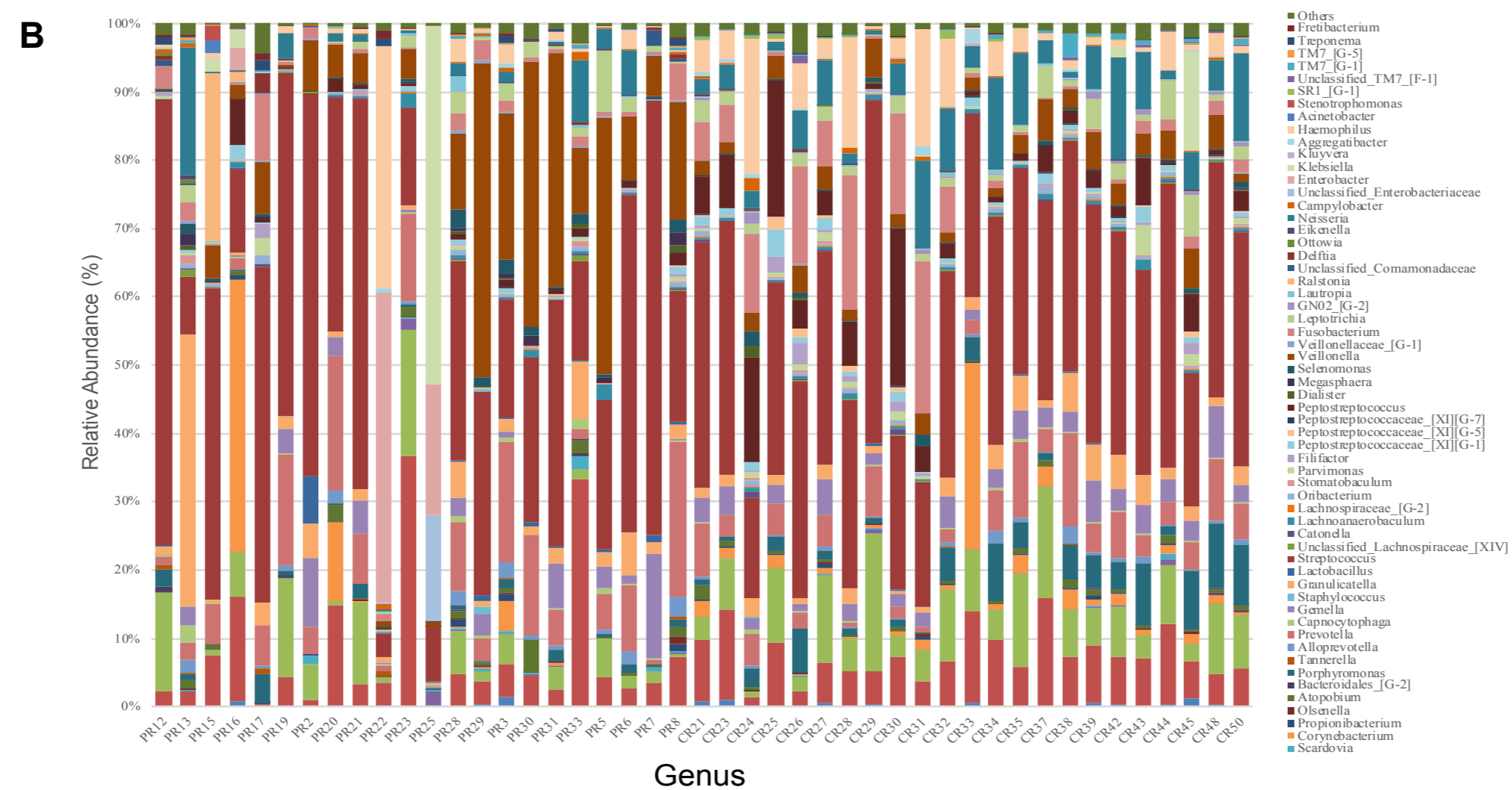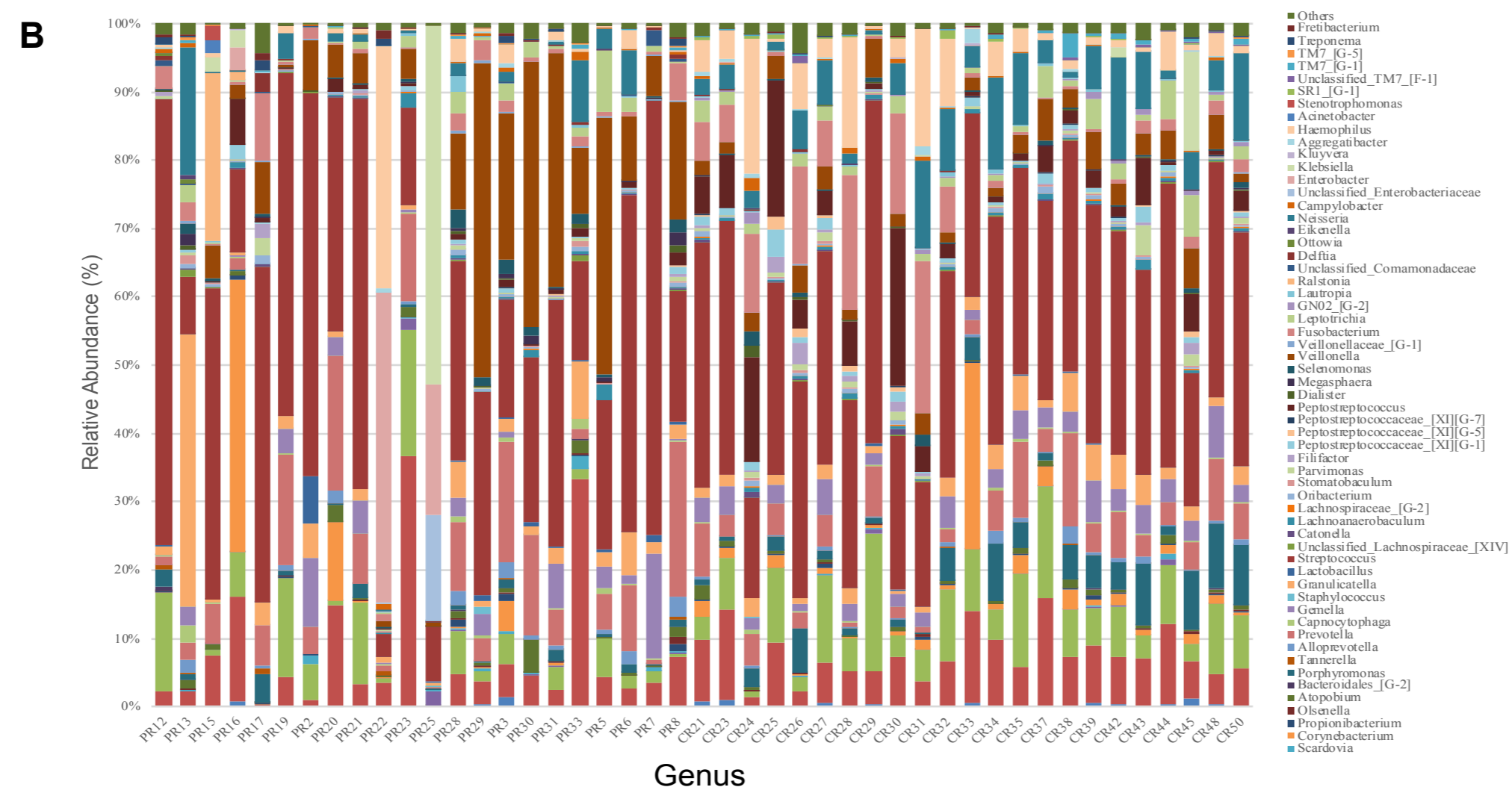

Supplement: Figure S1 — (A) At phylum level; and (B) at genus level. All the phyla and the 62 genera with relative abundance >1% are shown and listed. PR, rinse samples of pSS patients; CR, rinse samples of healthy controls. [file peerj-06-5649-s001.pdf]

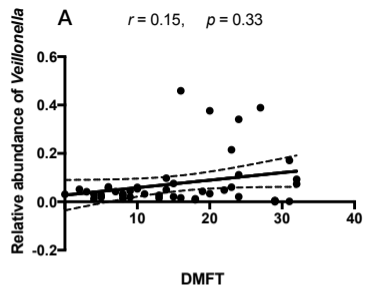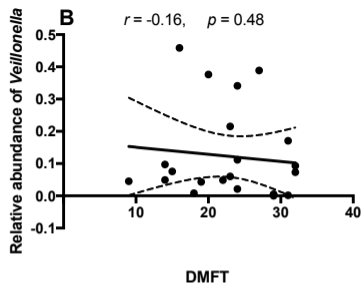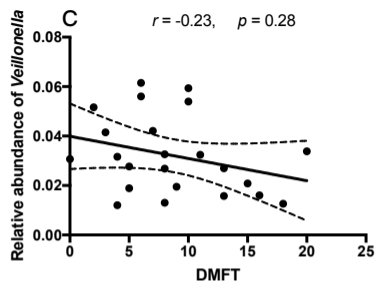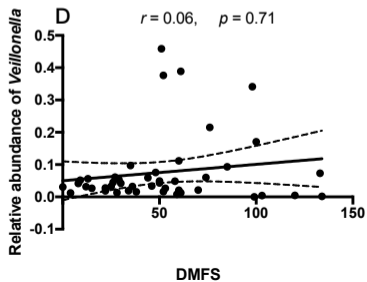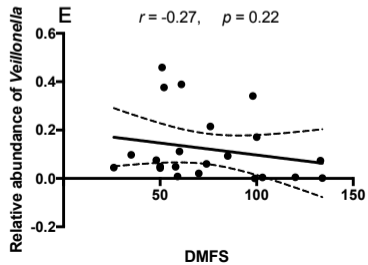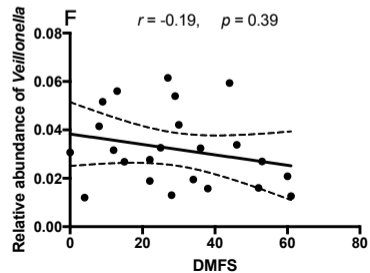

Supplement: Figure S2 [file peerj-06-5649-s002.pdf]
